# Involvement of Inner Choroidal Layer in Choroidal Thinning during Regression of Multiple Evanescent White Dot Syndrome

**DOI:** 10.1155/2019/6816925

**Published:** 2019-05-02

**Authors:** Yuki Hashimoto, Wataru Saito, Yuka Hasegawa, Kousuke Noda, Susumu Ishida

**Affiliations:** ^1^Department of Ophthalmology, Faculty of Medicine and Graduate School of Medicine, Hokkaido University, Sapporo, Japan; ^2^Kaimeido Eye and Dental Clinic, Sapporo, Japan

## Abstract

**Purpose:**

To investigate relationships between total thickness and the thickness of inner and outer layers in the choroid during regression in patients with multiple evanescent white dot syndrome (MEWDS).

**Methods:**

This retrospective observational case series included 15 unilaterally affected eyes and 13 unaffected fellow eyes from 15 MEWDS patients (4 men and 11 women; mean age, 37.6 ± 17.6 years). Using enhanced depth imaging optical coherence tomography, whole, inner, and outer choroidal layer thicknesses at the fovea and perifovea were manually measured at the initial visit and at 1 and 3 months after the initial visit. The mean thickness values of the layers were compared at each stage.

**Results:**

With regression of MEWDS, the mean subfoveal whole and inner choroidal layer thicknesses significantly decreased at 1 and 3 months compared to baseline values in MEWDS eyes (*P*=0.01 and *P* < 0.0001, respectively), but not in fellow eyes. The outer layer in MEWDS eyes tended to thin. Changes in the inner and outer layers at the perifovea in MEWDS eyes also showed the same trends. Simple linear regression analysis revealed significant positive correlations in choroidal thickness changes between the whole and inner layers (*R* = 0.53, *P*=0.04) and between the whole and outer layers (*R* = 0.91, *P* < 0.0001) from baseline to 3 months. Multiple linear regression analysis revealed that choroidal thickness changes in the whole layer were significantly correlated with those in the inner (*β* *=* 0.51, *P* < 0.0001) and outer (*β* *=* 0.73, *P* < 0.0001) layers.

**Conclusion:**

The inner choroidal layer significantly thinned with regression of MEWDS, correlating with the thinning of total choroidal thickness. These results suggest that MEWDS lesions in the choroid are likely to lie mainly in the inner layer.

## 1. Introduction

Multiple evanescent white dot syndrome (MEWDS) is an inflammatory chorioretinal disease associated with transient subretinal white dots. In eyes with MEWDS, optical coherence tomography (OCT) shows macular photoreceptor impairment, which is associated with visual function of MEWDS [1]. Hyperautofluorescence observed on fundus autofluorescence corresponding to the acute MEWDS lesions reveals the presence of retinal pigment epithelium lesions in this disease [2].

The etiology causing photoreceptor impairment in MEWDS is still unresolved. Recent observations on OCT angiography in MEWDS showed contradictory results (reduced or preserved flow void at the choriocapillaris) [3–6]. These results may indicate that the presence of circulation disorder at the level of choriocapillaris has yet to be determined in MEWDS. However, multiple hypofluorescent spots observed in MEWDS on indocyanine green angiography (ICGA) suggested choriocapillaris hypoperfusion [7]. During the regression of MEWDS, macular choroidal thickness significantly decreased and choroidal blood flow velocity derived mainly from the outer choroidal layer significantly increased, while these parameters correlated with visual function, suggesting a close link between choroidal abnormalities and visual impairment [8–10]. Recent layer-by-layer investigation into the choroid of 5 eyes with MEWDS demonstrated a nonsignificant tendency for both inner and outer layers to thin after onset [11]; however, statistical relationships between thickness changes in the whole choroid and each layer have yet to be clarified. Here, we sought to further analyze statistical relationships between thickness changes in the whole choroid and each layer during the course of MEWDS.

## 2. Methods

### 2.1. Patients

This retrospective observational case series included 15 unilaterally affected eyes and 13 unaffected fellow eyes from 15 MEWDS patients (4 men and 11 women). All patients were followed up with no treatment. The current study was approved by the ethics committee of Hokkaido University Hospital (#014-0182) and followed the tenets of the Declaration of Helsinki.

### 2.2. Ophthalmic Examinations

At the initial visit, each patient underwent thorough ophthalmic examinations including best-corrected visual acuity (BCVA), indirect ophthalmoscopy, fluorescein angiography, ICGA, 20 J single-flash electroretinography, and enhanced depth imaging optical coherence tomography (EDI-OCT) (RS-3000 Advance; NIDEK, Gamagori, Japan). BCVA and OCT findings were assessed at baseline as well as at 1 and 3 months after baseline.

### 2.3. EDI-OCT

**Figure 1:**
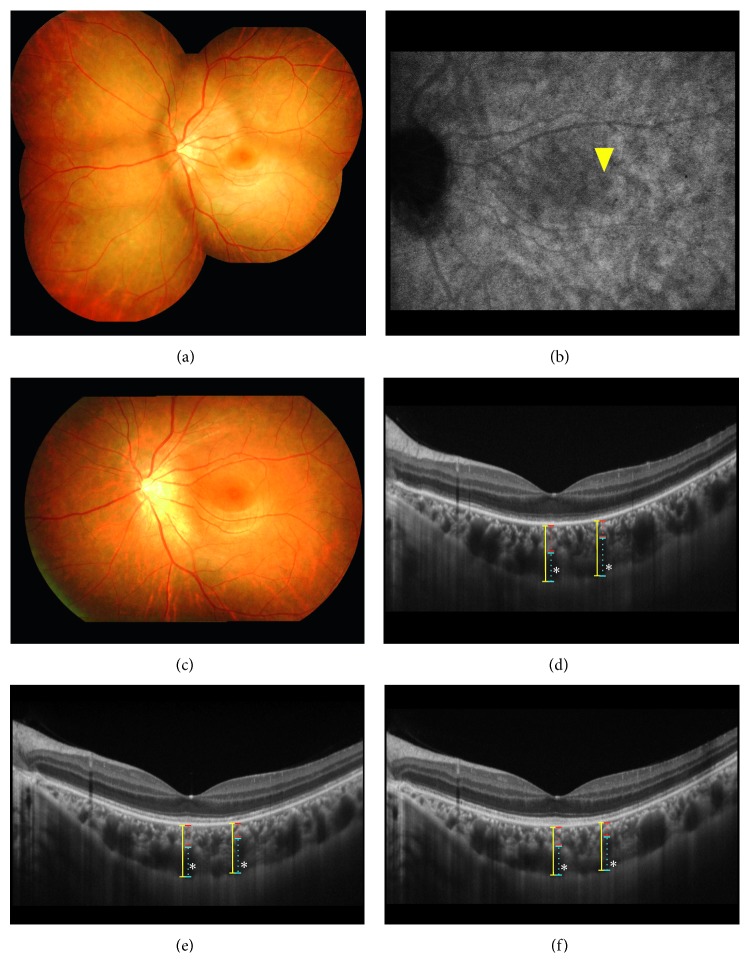
Images of the left eye in a patient with multiple evanescent white dot syndrome (MEWDS). (a) Funduscopic photograph showing multiple subretinal white dots extending from the posterior pole to the midperiphery and foveal granularity at the initial visit. (b) Late-phase indocyanine green angiograpahy (ICGA) showing numerous hypofluorescent spots scattered more broadly than white dots. An arrowhead indicates the closest hypofluorescent spot from the fovea where choroidal thickness was measured, as shown in Figure 1(d). (c) Three months after the initial visit, the white dots spontaneously resolved. (d–f) Horizontal enhanced depth imaging optical coherence tomography images through the fovea. The outer choroidal layer thicknesses (blue lines) were measured from the inner border of the choroid-sclera junction to the innermost points of large choroidal vessels (asterisks) observed in closest proximity to the subfovea and the perifovea (identical with an arrowhead of Figure 1(b)). The inner choroidal layer thicknesses (red lines) were obtained via subtraction of the outer layers (blue lines) from the whole thicknesses (yellow lines). Diffuse loss of the ellipsoid zone at the macular area was observed at the initial visit, with subfoveal and perifoveal lesions (Fig.  1(b); yellow arrowhead) thickness values of 146, 237, and 384 *µ*m and 111, 264, and 375 *µ*m for the inner, outer, and whole layers, respectively (d). One month after the initial visit, the macular ellipsoid zone improved. Thickness values at the subfoveal and perifoveal lesion sites decreased to 133, 225, and 359 *µ*m and 99, 243, and 342 *µ*m in the inner, outer, and whole layers, respectively (e). Three months after the initial visit, thickness values at these lesions further decreased to 122, 207, and 330 *µ*m and 95, 235, and 330 *µ*m in the inner, outer, and whole layers, respectively, with spontaneous complete recovery of the macular ellipsoid zone (f).

Using both horizontal and/or vertical scans (scan length, 9.0 mm) through the fovea of EDI-OCT, we manually measured choroidal thicknesses of whole, inner, and outer layers at the subfovea with granular changes and the closest one or two lesions (i.e., a white dot and/or a hypofluorescent spot on ICGA (Figure 1(b))) from the fovea, as described previously [12]. Regarding the measurements at the perifovea in each case, we adopted a single measurement result if the lesion was observed only in one image from the horizontal and vertical scans. In total, we could perform choroidal thickness measurements of 15 (all horizontal images) at the fovea and 25 (13 horizontal and 12 vertical images) at the perifovea. Measurements were collected at baseline, 1 and 3 months in MEWDS eyes, and at baseline and 3 months in fellow eyes. We determined the statistical significance of the differences in average thickness values of each layer between stages.

### 2.4. Statistics

The Friedman test and Scheffe's paired comparison test or Wilcoxon's signed-rank test were used to compare changes in logMAR BCVA and choroidal thicknesses in the whole, inner, and outer layers. Spearman's rank correlation test was used to determine relationships between the rate of change in thickness for the whole layer and that for the inner and outer layers. A multiple regression analysis determined independent variables affecting thickness changes in the whole choroidal layer. For all tests, *P* < 0.05 was considered statistically significant.

## 3. Results

### 3.1. Patient Demographics

The mean age was 37.6 ± 17.6 years ranging from 16 to 74 years. The mean follow-up duration was 18.4 ± 11.4 months ranging from 3 to 40 months. All patients had unilateral involvement at the initial visit, and no unaffected fellow eye developed signs of acute zonal occult outer retinopathy during follow-up. The mean refractive error was −5.5 ± 3.8 *D* ranging from +1.5 *D* to −12.0 *D* in MEWDS eyes and −5.4 ± 3.5 *D* ranging from +1.0 *D* to −11.0 *D* in unaffected fellow eyes. There was no significant difference in refractive error between the two groups (*P*=0.91).

### 3.2. Ophthalmic Findings

In all MEWDS eyes, subretinal white dots (Figure 1(a)) spontaneously resolved within 1 month after the initial visit (Figure 1(c)) with recovery of the macular ellipsoid zone (Figures 1(d)–1(f)). The mean logMAR values of BCVA at baseline and 3 months after baseline were 0.39 ± 0.44 and −0.01 ± 0.18, respectively, demonstrating significant improvements at 3 months compared with the baseline value (*P* < 0.0001). No affected eyes developed ocular complications including choroidal neovascularization.

### 3.3. Changes in Choroidal Thickness

**Figure 2:**
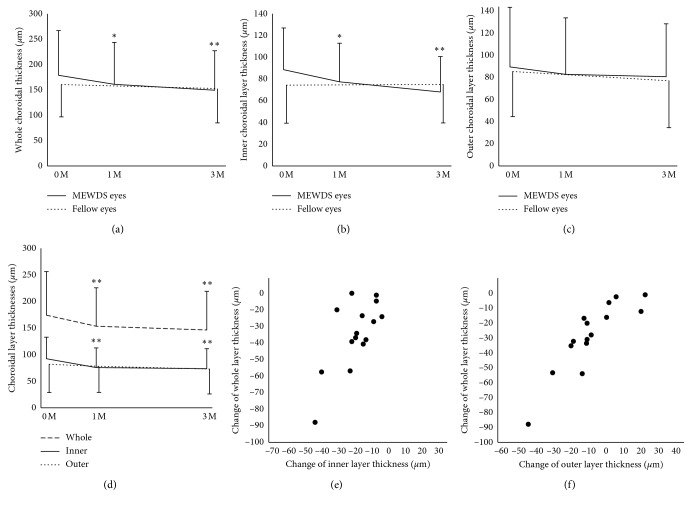
Sequential changes in choroidal thickness of the mean whole, inner, and outer layers at the subfovea (a–c) with granular changes and the perifovea (d) with a white dot and/or a hypofluorescent spot observed on ICGA in patients with MEWDS, and correlations in choroidal thickness change from baseline to 3 months between the whole and inner layers (e) and between the whole and inner layers (f) at the subfovea in MEWDS eyes. (a) The mean whole choroidal thickness in MEWDS eyes (*n*=15, black line) significantly decreased at 1 and 3 months, compared to baseline (Friedman test, *P* < 0.0001; Scheffe's paired comparison test, *P*=0.01 and *P* < 0.0001, respectively). By contrast, there was no significant change in the whole thickness of fellow eyes (*n*=13, dotted line, Wilcoxon signed-rank test, *P*=0.08). (b) The mean inner choroidal layer thickness in MEWDS eyes significantly decreased at 1 and 3 months, compared to baseline (Friedman test, *P* < 0.0001; Scheffe's paired comparison test, *P*=0.01 and *P* < 0.0001, respectively). By contrast, there was no significant change in the inner layer thickness of fellow eyes (*P*=0.64). (c) The mean choroidal outer layer thickness in MEWDS eyes tended to reduce with no statistically significant difference during the 3-month follow-up period (Friedman test, *P*=0.12). There was no significant change in the outer layer thickness of fellow eyes (*P*=0.16). (d) Similarly, the mean whole and inner choroidal thicknesses (*n*=25) in MEWDS eyes significantly decreased at 1 and 3 months, compared to baseline (Friedman test, *P* < 0.0001 for each; Scheffe's paired comparison test, *P* < 0.0001 for each and *P*=0.0002, *P* < 0.0001, respectively). The outer choroidal layer thickness in MEWDS eyes tended to reduce (Friedman test, *P*=0.13). (e, f) There were significant positive correlations in baseline-to-3-month thickness changes between the whole and inner layers (Spearman's rank correlation coefficient, *R* = 0.53, *P*=0.04) (d) and between the whole and outer layers (*R* = 0.91, *P* < 0.0001) (e).

The mean whole choroidal thicknesses at the subfovea and the perifovea in MEWDS eyes significantly decreased at 1 and 3 months, compared to baseline (Figures 2(a) and 2(d); Friedman test, *P* < 0.0001 for each; Scheffe's paired comparison test, *P*=0.01, *P* < 0.0001, and *P* < 0.0001 for each, respectively). Similarly, the mean inner choroidal layer thicknesses at both of these lesions in MEWDS eyes significantly decreased at 1 and 3 months, compared to baseline (Figures 2(b) and 2(d); Friedman test, *P* < 0.0001 for each; Scheffe's paired comparison test, *P*=0.01, *P* < 0.0001 and *P*=0.0002, and *P* < 0.0001, respectively). However, the outer choroidal layer thicknesses at the subfoveal and perifoveal lesions in MEWDS eyes tended to reduce with no statistically significant difference during the 3-month follow-up period (Figures 2(c) and 2(d); Friedman test, *P*=0.12, *P*=0.13, respectively). In fellow eyes, subfoveal choroidal thicknesses in the whole, inner, and outer layers were unaltered throughout the follow-up (Wilcoxon signed-rank test, *P*=0.08, *P*=0.64, *P*=0.16).

### 3.4. Correlation between Whole and Inner or Outer Choroidal Thicknesses

Simple linear regression analyses revealed significant positive correlations in choroidal thickness changes between the whole and inner layers at the subfovea and the perifovea (Figure 2(e); *R* = 0.53, *P*=0.04 and *R* = 0.49, *P*=0.01, respectively) and between the whole and outer layers (Figure 2(f); *R* = 0.91, *P* < 0.0001 and *R* = 0.73, *P* < 0.0001, respectively) from baseline to 3 months. Multiple regression analyses revealed that choroidal thickness changes in the whole layer at the subfoveal and perifoveal lesions were significantly correlated with those in the inner (*β* *=* 0.51, *P* < 0.0001 and *β* *=* 0.61, *P* < 0.0001, respectively) and outer (*β* *=* 0.73, *P* < 0.0001 and *β* *=* 0.53, *P* < 0.0001, respectively) layers, but not with other clinical parameters including visual acuity, age, or refraction.

## 4. Discussion

In the present study, the mean macular whole and inner layer choroidal thicknesses significantly decreased with regression of MEWDS, while the outer layer did not. However, a multiple linear regression analysis revealed that choroidal thickness changes in the whole layer showed significantly positive correlations with those in both the inner and outer layers.

In the present layer-by-layer analyses, a significant difference in choroidal thickness reduction was detected in the inner rather than outer choroid in MEWDS eyes. In patients with Vogt–Koyanagi–Harada (VKH) disease, we previously showed that choroidal thinning during the initiation of systemic corticosteroid therapy depends on outer but not inner layer thickness changes, suggesting that the choroidal outer layer is primarily affected in the acute stage of VKH disease [13]. Thus, major foci in the choroid were different between eyes with MEWDS and VKH disease, although both are considered as inflammatory diseases with choroidal thickening in the acute stage.

In simple linear and multiple regression analyses, choroidal thickness changes in the whole layer exhibited significantly positive correlations with those in both the inner and outer layers, although the outer layer failed to show a statistically significant reduction. This may be attributable in part to the small number of patients enrolled in the present study and the inherent thinness of the myopic choroid as usually seen in MEWDS. Indeed, choroidal blood flow velocity derived mainly from the outer layer significantly increased with regression of MEWDS [9]. Taken together, MEWDS lesions in the choroid are likely to lie mainly in the inner layer. The present study showed no apparent evidence regarding impact of the outer layer on the total thickness. Further studies are needed to investigate the role of the outer layer in MEWDS.

As concerns the mechanism underlying the increase of choroidal layer thicknesses in MEWDS eyes at the acute stage, two possibilities are speculated as follows: (1) the progression of outer retinal inflammation into the choroid; (2) the foci of MEWDS lesions at the choroid initially. Recent studies showed hyporefrective lesions at the level of the photoreceptors on en face OCT with flow preservation at the choriocapillaris on OCT angiography corresponding to the white dots, suggesting the photoreceptor is primarily affected in MEWDS [3, 4]. However, others showed a few reports showing reduction of the flow void at the choriocapillaris on OCT angiography corresponding to part of white dots in MEWDS [5, 6]. As concerns these contradictory results, the current OCT angiography modalities may not so sensitively detect circulatory disturbance at the choriocapillaris in MEWDS, because the extent of circulatory disorders at the choriocapillaris in MEWDS is milder than that in other white dot syndromes, as observed on ICGA [7]. In white dot syndromes other than MEWDS (e.g., acute posterior multifocal placoid pigment epitheliopathy, birdshot chorioretinopathy, and punctate inner choroidopathy), indeed, OCT angiography demonstrated choriocapillaris flow reduction, suggesting that lesions in these diseases primarily affect the choroid [14]. Therefore, mild flow reduction at the choriocapillaris that could not be detected by OCT angiography may be sufficient to cause outer retinal changes as MEWDS lesions. Still, we believe that the origin of MEWDS lesions is a subject of investigation.

The present study has some limitations. This study is a retrospective design with relatively small number of patients. We manually measured choroidal layer thicknesses using OCT B-scan; thus measurement bias would not be denied.

## 5. Conclusions

In the present study, the mean macular whole and inner choroidal layer thicknesses in the affected eyes significantly decreased with regression of MEWDS. However, the outer layer did not significantly thin. Moreover, choroidal thickness changes in the whole layer exhibited significantly positive correlations with those in both the inner and outer layers. The current results suggest that choroidal lesions in MEWDS lie mainly in the inner layer.

## Data Availability

The data used to support the findings of this study are available from the corresponding author upon request.
